# A New Medical School in Glasgow

**Published:** 1875-11

**Authors:** 


A New Medical School in Glasgow.—The Glasgow Royal Infirmary is about to receive a charter as a medical school. The school already exists, and is said to be in a highly-flourishing condition as to students and facilities for teaching them.—*New York Medical Journal.*

